# Schools at the center of a public health emergency: leveraging schools for pediatric COVID-19 vaccination

**DOI:** 10.3389/fpubh.2023.1185878

**Published:** 2023-06-08

**Authors:** Sara Bode, Mary Kay Irwin, Hannah Palme', Daniel Skinner

**Affiliations:** ^1^School Health Services, Nationwide Children's Hospital, Columbus, OH, United States; ^2^Heritage College of Osteopathic Medicine, Ohio University, Dublin, OH, United States; ^3^Department of Social Medicine, Heritage College of Osteopathic Medicine, Ohio University, Dublin, OH, United States

**Keywords:** vaccination, COVID-19, adapting, public health, school-based health care, Ohio (U.S.A.), children's hospital

## Abstract

The U.S. Food and Drug Administration's expansion of COVID-19 vaccine eligibility in 2021 to include children presented opportunities and challenges to ensure widespread access. Children, and especially adolescents, were a crucial target population to reduce community positivity rates and support a resumption of in-person academics. Though existing school-based vaccination programs have demonstrated success in improving vaccination rates on an individual school level, best practice strategies for employing mass vaccination programs quickly in response to public health emergencies have yet to be identified. Through established partnerships, School Health Services at Nationwide Children's Hospital led a collaborative effort to employ a rapid, onsite school vaccination strategy across Franklin County for all eligible students. This collaboration resulted in a significant increase in vaccine access carried out through on-site vaccination clinics established in 20 local public and private school districts. Key strategies identified through the process included collaboration with school districts, local hospitals, and the public health department; calibrating program size to each site and number of vaccines needed; and coordination of team member roles. At the same time, experience with the effort also underscored key challenges and opportunities that future programs should consider, especially when operating in public health emergencies. School-based community health approaches targeting adolescents can increase vaccination rates, and can be successfully led by children's health systems in concert with public health departments and schools. At the same time, entities undertaking such efforts must plan in advance to ensure that partnerships can be effectively established with clear protocols for efficient and open communication, which is essential for overcoming barriers in access to healthcare services.

## Introduction

Though a great deal has been written about the experience of the early months of the pandemic and what those months taught us about public health in the U.S., we have only just begun to understand how we should use these lessons to better prepare for the next public health emergency. This is particularly true regarding structure and preparedness within American kindergarten through 12th grade (K-12) education systems, utilizing schools as a central component of the public health response. School-located vaccine programs, particularly for influenza, have shown effectiveness in overall increased vaccination rates across communities, making this a key component of the public health response (1).

In July 2021, the Biden administration called on schools to host COVID-19 vaccination clinics for children as a critical strategy to improve vaccination rates and maintain in-person learning for students (2). The Department of Health and Human Services, in collaboration with the Centers for Disease Control, produced a School Communities toolkit with planning considerations for school vaccine clinics (3, 4). In Ohio, as elsewhere, the pandemic shined a light on a dedicated and committed workforce. At the same time, no comprehensive assessment of what we learned from the pandemic has been undertaken by the state, leaving the task to individual organizations and scholars (5).

In this perspective we describe a mass pediatric vaccination effort undertaken by School Health Services at Nationwide Children's Hospital (SHS) in Columbus, Ohio. We begin with the initial positioning SHS had in Central Ohio prior to the pandemic's arrival in March of 2020, including important opportunities within the education system. We also address challenges to vaccine mobilization and distill key lessons learned from this analysis that could prove useful for addressing future pandemics and public health emergencies.

## Timeline

To appreciate the challenges presented by the pandemic's second year, when vaccinations first began to become available for children, we must first take stock of the major developments leading up to and during that early period. In March 2020, the first cases of COVID-19 became known to Ohio public health departments and healthcare institutions. This led to an ongoing, changing response from state leadership to mobilize an infection control response as public health experts' understanding of the pandemic progressed. The whiplash of the moment can be captured by Governor Mike DeWine's March 12, 2020 announcement that schools would be closed for a “period of several weeks,” (6) only to be amended a month later, on April 20, that those same schools would remain closed for the rest of the academic year (7). Advisories from the state would be amended on an almost weekly–and sometimes daily–basis.

By July 2, just a month after the governor highlighted a rapid increase in cases infecting children, he announced that schools would return to in-person sessions in the fall, with a wide range of COVID-19 precautions–reflecting available resources and infrastructure conditions–informed by a return to school manual (8). This manual was followed up on August 4 with a joint statement issued by the Governor's Office, the American Academy of Pediatrics, and the Ohio Children's Hospital Association reasserting the importance of best practices–including the wearing of masks–for Ohio schools (9, 10). The governor noted, “Without a vaccine, we are limited in the ways that we can protect the people of Ohio,” adding that “For schools to have a fighting chance to stay open this fall, widespread face coverings for K-12 students will increase the odds that kids will go to school and stay in school.”

This iterative public health response would remain the norm for the rest of 2020 and would continue into 2021. In many ways, however, the full mobilization of Ohio's pediatric and public health communities would be placed in something of a holding pattern until vaccines became available. As is now well-documented, the arrival of vaccinations for adults in late 2020, and older children in early 2021, came not only with a sense of relief, but intense politicization. This was evident through state press conferences and news coverage that highlighted the concerns for vaccine mistrust in the community, often led by targeted vaccine misinformation (11).

And yet, as health experts in Ohio well knew, it would take a full-scale mobilization to begin vaccinating Ohio's pediatric population once approved for use in children and adolescents. This work would be all the more challenging considering the growing amount of mistrust due to misinformation circulating the state.

**Figure 1:**
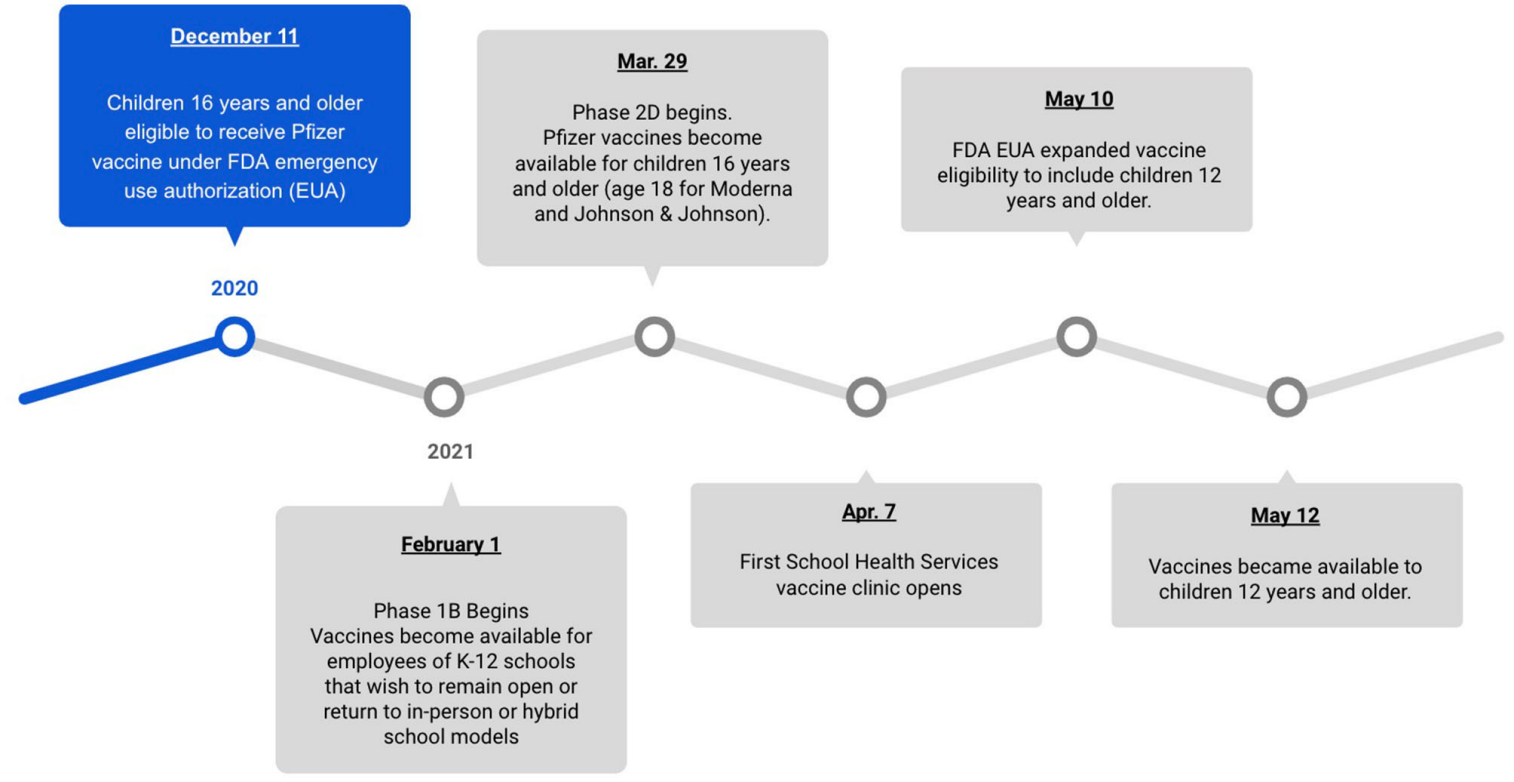
Vaccination key dates.

A period known as Phase 2D commenced on March 29, 2021 when vaccines were authorized by the U.S. Food and Drug Administration for children ages 16 and older (see Figure 1). As vaccines became available, it became immediately apparent that a novel strategy focused on the adolescent population would be necessary to ensure access across the county. Although existing partnerships with schools existed, they were not intended for mass pandemic vaccine mobilization; however, SHS was able to quickly facilitate a collaboration with the local public health department and area school districts to execute a mass COVID-19 vaccination strategy for adolescents across Franklin County.

## Transforming existing partnerships

For SHS, the goal of Phase 2D was to use existing partnerships with schools to mobilize a vaccination program before the end of the academic school year in May. Predictably, the arrival of this phase came with opportunities as well as challenges.

The opportunities for children's hospitals were clear. While entities from public health departments, hospitals, and health care systems mobilized in various ways across the state, children's hospitals were uniquely positioned to facilitate this process. Because of pre-pandemic groundwork, SHS was able to use existing health partnerships to drive a pediatric-focused vaccine strategy using long-established SHS relationships with Central Ohio schools, allowing access to children directly at the schools they attended. It was essential for SHS vaccine mobilization to act quickly due to the uncertainty of school closures as waves of COVID-19 outbreaks resurfaced and the end of the academic school year approached.

**Figure 2:**
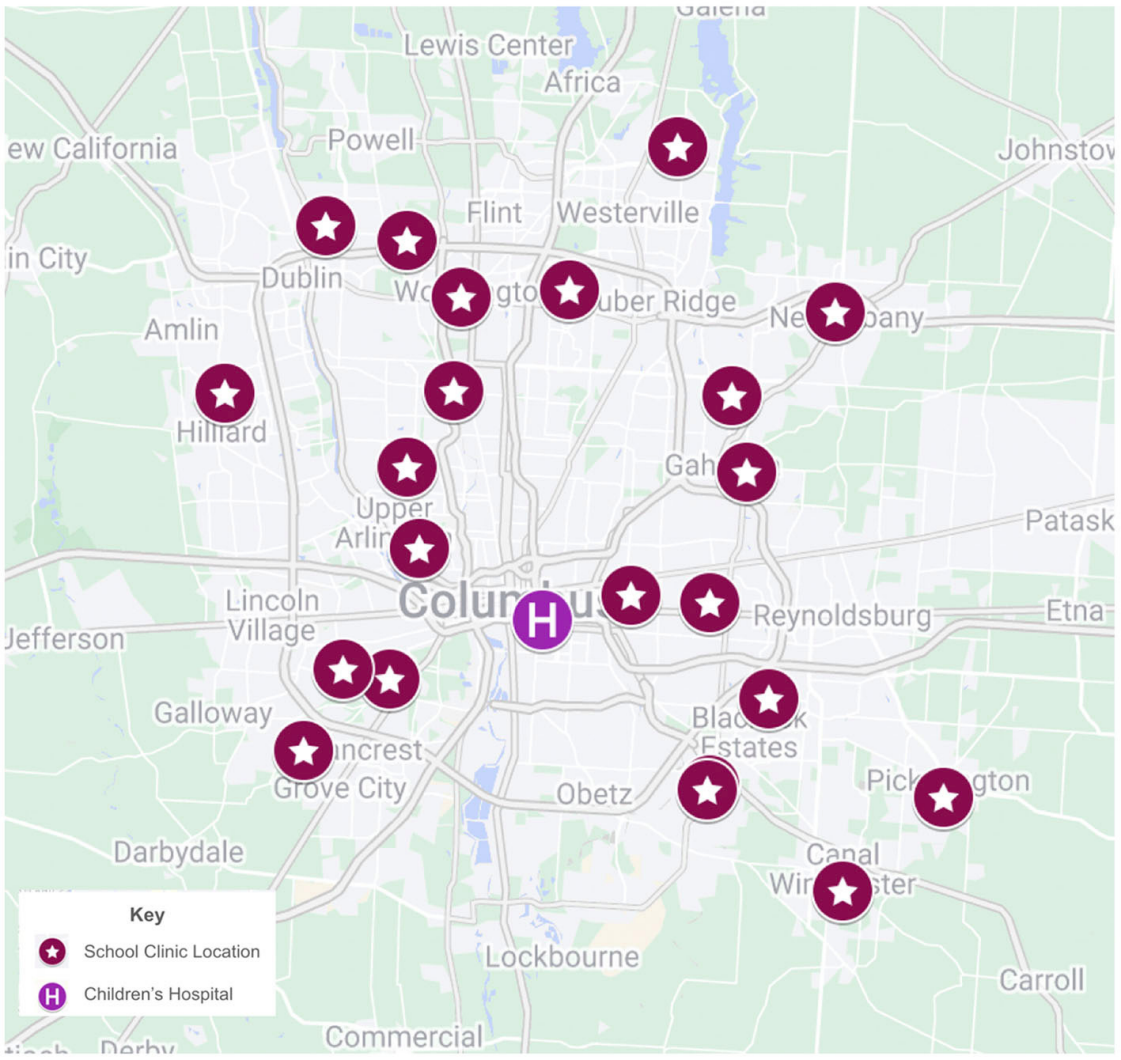
Phase 1 vaccine clinic geography.

For many years, SHS has worked with key community stakeholders to address the needs of children and families in order to provide equitable health care. As part of these efforts, SHS's existing partnerships began with school-based behavioral health services and two mobile health care units delivering comprehensive primary care at community locations including schools, and early childhood centers. By the time vaccines arrived in 2021, SHS partnerships included 14 school-based health centers (SBHC), robust mobile medical care, and the associated chronic disease programs serving students in 15 public districts in the county (see Figure 2). Though pandemic preparation was not part of the original design for SHS's programming, the presence of preexisting school health relationships provided a foundation to build from during emergencies.

## “The 6 week sprint”: planning, communication, and implementation of the program

**Figure 3:**
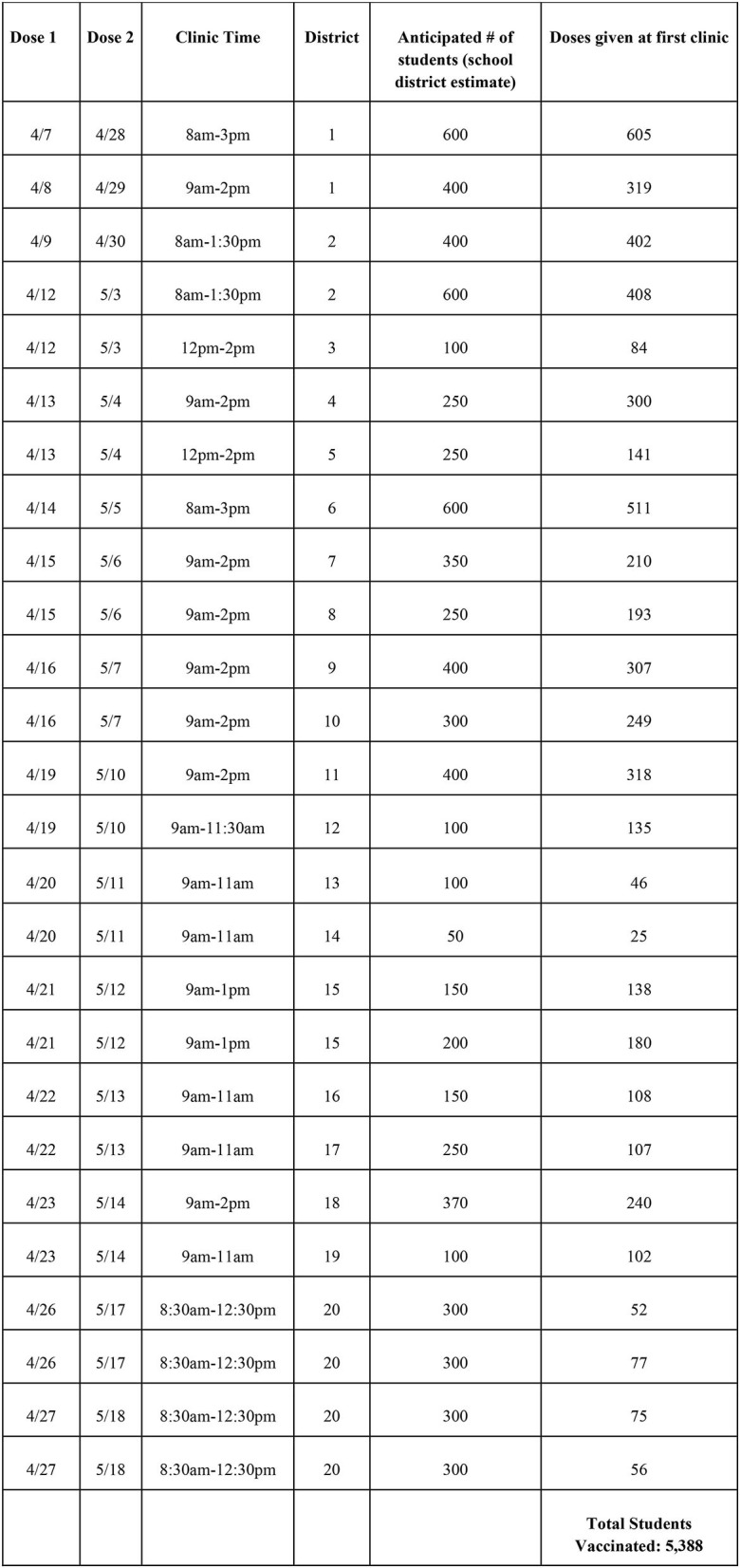
“6-week sprint” of clinics for the adolescent population. April 7, 2021–May 26, 2021.

As mentioned, March 29, 2021 started phase 2D which allowed for COVID vaccination of children ages 16 years and older. This led to a very short timeline for administering the recommended two doses of the COVID vaccine to adolescents prior to the end of the school year. In order to implement a county-wide vaccination program for adolescents, it was determined that clinics would need to begin the week of April 5, 2021. This would allow three full weeks to administer the first dose of COVID vaccine with all 20 school-district partners, with plans for an additional 3 weeks for second dose administration prior to the end of the academic year (see Figure 3). Planning for the adolescent COVID-19 vaccine project consisted of coalition building with several partners including school personnel, local families, local public health departments, and health care systems. The initial outreach, to discuss capacity building through a partnership between SHS and area schools, came from the commissioner of Columbus Public Health, the city's health department, to each school district the week of March 22, 2021. Existing school and community partnerships with many local school districts was a key component to rapidly forming this coalition with the goal to complete both doses of vaccination before the end of the 2020–2021 academic year for each school district.

The first steps in anticipation of phase 2D of COVID-19 vaccination rollout included discussion among the city, county, and state public health departments, and the area adult health care systems to determine the availability of vaccines. At the time, vaccine availability was tenuous, with an uncertain guarantee of allotment per week to each healthcare system. Due to scarcity, local health departments and adult health systems each agreed to transfer portions of their vaccine allocation as a coordinated effort to improve access for pediatrics, particularly in the first weeks of the program. In time, the Ohio Department of Health began to provide vaccines directly to Nationwide Children's Hospital. Following confirmation that vaccines were procured, outreach to public school district superintendents began and with approval, the SHS team worked directly with each district's primary point of contact to determine location, scheduling, and estimating probable demand. Communication with each district was led by our SHS team, specifically our director of partnerships. Each district had representation including the Superintendent, the Principal at the chosen vaccine site, the school nurse, and a parent representative. This team determined for each district how the consent forms would be distributed, what school communication channels were best (i.e., recorded calls to families, email, text messaging, social media outreach), where the clinic would be located in the building, and what school staff would be responsible for traffic flow, transportation, and fielding questions from families and students.

A SHS program manager and marketing specialist worked with each district's point of contact and communication team to share marketing materials that provided vaccine clinic details and to educate the community by dispelling misinformation. School districts utilized the materials and individualized their outreach as appropriate. One high school from each district was chosen as the clinic site, with the exception for the largest district in the county which had four locations to accommodate its 20 high schools. Factors determining site selection included central location, outdoor access to the school gymnasium, as well as schools with the highest percentage of students experiencing barriers to accessing health services based on federal Title I designation under the Elementary and Secondary Education Act, which signifies that schools serve high numbers of students from low-income families (12). Specifically, across the school districts with which SHS works there are certain facilities that serve a higher percentage of low-income families, and these facilities were prioritized in the location selection for each district. As these districts report disparities across their high schools, we chose the high school within the district that would prioritize more of these students. The program manager provided each point of contact with a physical clinic set-up guide to ensure that details were not overlooked.

The existing roving vaccine clinic process was utilized as the basis for the development of a mass vaccination process, with the flexibility to scale up and down depending on the size and needs of each district. Defined roles for the COVID-19 vaccine clinic included registration staff, vaccinators (registered nurses and advanced practice nurses), pharmacists, pharmacy technicians and interns, clinical observers, and school liaisons. Staffing needs were determined the week prior and scaled depending on survey results designed to assess demand and consents obtained before each clinic, including liberty for day-of additional patients. All partners agreed on one consent process and incorporated a verbal consent to increase vaccination efforts. Due to vaccine scarcity, remaining vaccine doses at the end of each clinic day were offered to school staff and community members to reduce waste. If doses remained, they were transported back to Nationwide Children's to be used in an onsite clinic.

A total of 20 school districts were scheduled for clinics with the ability to serve between 100 and 800 students per site (1–2 clinics operated Monday through Friday over the 6 weeks). A minimum of one clinic was offered in each public district and every private or charter school seeking vaccines. In total, 20 first and second-dose clinics were offered in 10 districts. These 20 clinics were operationalized in an intense 6-week effort (known in-house as the “6-Week Sprint”) in which first doses were distributed in the first 3 weeks, and then a second dose over the next three. Transportation was coordinated using school buses to transport students from high schools without onsite clinics to the closest clinic within their respective district (or area in the case of private and charter schools) and to time the arrival of each bus according to the number of doses that could be provided in defined segments of time. A typical clinic day consisted of set up at 7 am in a large, shared school space, typically the gymnasium, with clinic hours varying depending on volume, the longest being from 8 am−3 pm. Transportation from surrounding schools in the district were staggered throughout the day to provide for a consistent flow of students arriving to check in. Setup included three front check in desks, 10 vaccine tables complete with all vaccination materials and laptops for the provider to document and administer the vaccine, a waiting area for students to stay for the observed 15 min after vaccination, and a triage area for anyone needing further assistance with any anxiety or post vaccination symptoms. Clinics concluded by 3 pm at the latest each day to allow for transport back home through the regularly scheduled school process (see Figure 3).

## Discussion: lessons learned and preparing for the next pandemic

Vaccination in school-age children reduces illness, promoting daily attendance and increasing access to in-person learning opportunities and participation in extracurricular activities. The result of this increased access includes a direct effect on children's learning achievements, linking vaccination directly to improved educational outcomes (13). In addition to academic success, consistent in-person learning and extracurricular activities promote social-emotional growth, mental health, and overall wellbeing, which was negatively affected throughout the pandemic (14). Given these positive outcomes, universal vaccination promotion and access is a key strategy during a pandemic response. However, children eligible for COVID-19 vaccination may experience barriers to access including reliable transportation and required guardian participation. Additionally, they may experience vaccine hesitancy due to limited opportunity to engage with medical providers to offer vaccine education, answer questions, and dispel myths (15). School-based vaccination clinics are uniquely poised to employ strategies to address these vaccine barriers and provide education, making them an ideal location for large-scale mobilization during a pandemic.

While the school-based vaccination clinics described were largely successful, there were also challenges. One major challenge included the changing landscape of vaccine availability in the early planning stages of the clinics. The uncertainty of vaccine supply capacity led to an initial conservative estimate of children that could be served by these clinics. This meant that early communication with each school district could not guarantee vaccine supply in the moment. School districts had to move forward with communicating with parents and staff with the hope that these clinics would have available vaccines the day of the clinic. This took a level of trust in the partnership and the commitment from the health department that vaccines would be procured. An additional challenge included estimating the number of doses needed for each clinic in a short time frame. As seen in Figure 3, districts varied in the accuracy of estimating the number of students that would return consent and desire vaccination. Each district implemented different processes to determine this estimate including parent online surveys, student surveys, online consent forms, or school leadership estimates. This led to concern about vaccine waste and availability each day in clinics. A partnership with the main NCH hospital was needed to return unused doses quickly to be used in evening and next day vaccine clinics for adults and children to minimize any waste. Additional staff were also needed to courier vaccines to the school sites as needed for additional doses. Vials of vaccine that were thawed for administration and could not be returned to main campus were offered to school staff and parents at the end of each clinic to again minimize any wasted doses.

As would be expected in any large-scale mobilization, a series of challenges may complicate vaccine rollout efforts. We note four additional broad areas where challenges arose and lessons were learned:

1. Partnerships: Community partnerships take intentional efforts to build and sustain and are not easily formed during an emergency. Advanced planning, especially in forming partnerships, must begin early. This advanced planning consists of establishing a relationship between the school district and the health care partner to discuss potential areas of collaboration and clinical services during an emergency. Ongoing meetings discussing role delineation within school and health personnel, scope of services, potential space utilization, flow of communication are all critical components in planning for emergency services. This speaks to the core components of authentic community engagement, including establishing relationships, building trust, working with leadership, and seeking commitment from community organizations to create processes for mobilizing the community (16). Working with communities ensures that the population impacted by the problem is involved in co-creating solutions to ensure shared power with decision making. Establishing longitudinal relationships within the community and listening to members can build trust, which lays the foundation to support community-led solutions in times of crisis (17).
Due to the quick timeline from initial partnership to vaccine implementation, several challenges arose with some school districts. If the SHS team did not have an existing partnership it took much longer to get in contact with the correct team members to lead the initiative at the district. This led to a shorter timeline for the school to communicate with their students and families and affected vaccine consent and uptake for some districts. In some cases, students and parents were notified about the potential vaccine opportunity only 2 or 3 days prior to the first clinic. Those families were also not familiar with the SHS team and therefore may have chosen to defer the vaccine at that time. Pre-planning for future emergency plans with each district will be a valuable step moving forward.
2. Communication: Throughout the mobilization process, consistent, clear, and intentional communication for each unique community was a key component to the success of these clinics. To start, this included a clear shared vision regarding scope of initiative and target population to be served. Weekly zoom calls coupled with email communication sharing best practices, vaccine product needs, and successes and challenges with each community were critical. Another layer of essential communication included leadership and community representation from each school district to determine how best to meet the needs of each community. Finally, and most importantly, was ongoing clear communication (e.g., focus groups to tailor messaging, virtual town halls for Q & A, dissemination of written materials addressing facts and myths, social and television media messaging highlighting student and parent voice) with students and families to spread awareness of the opportunity, provide evidence-based information, and to dispel myths and misinformation. This was easily implemented with school district's that had an existing relationship with the SHS team and had utilized these communication channels in the past on varying health topics and messaging. Established health advisory groups, previous town halls throughout the pandemic, a partnership between hospital and school district marketing all led to improved deployment of the messaging and communication. This did not occur in depth with districts that did not have an existing partnership, however it was beneficial to have the weekly Zoom calls for each district to hear about the existing frameworks in place in some schools to stand up a communication plan as quickly as possible.
3. Planning: The utilization of existing protocols and systems were an essential part of the vaccine mobilization effort. Due to pre-existing school health services, which included both health care delivery in school-based health centers as well as roving immunization clinics, a process was already established for implementation of large-scale school-based COVID vaccine clinics. This included established checklists and protocols for assessing clinic logistics and infrastructure such as physical space needs, ideal locations within schools, Wi-Fi/power needs, and transportation. Examples of lessons learned included the need for advanced assessment of available physical space such as school gymnasiums, inclusive of space, patient flow, as well as power outlets, extension cords, and needed hot spots for areas with poor Wi-Fi signal. Planning was also critical for timing of transportation from school to school to maintain good clinic flow and utilize start and end times of the school day for vaccinating onsite students and staff. Resources and staff to support outreach, community education, clinic flow, and follow-up were quickly identified for both the clinical and school teams. This included clear roles and responsibilities for each team member and daily huddles for day of communication. This established clinical line of services benefitted all district partners due to a clear, replicable clinic structure and protocol.
4. Access to care: SHS traditionally partners with communities that have identified gaps in comprehensive healthcare utilization. Providing additional access to care where students spend the majority of their day allows for a true population health approach (18). This paradigm remained true for delivery of the COVID-19 vaccine. Centralized hospital located vaccine clinics served as an important strategy for vaccine dissemination but did not meet the need for all families with barriers such as transportation, health literacy, and online scheduling access. By utilizing existing school partnerships, COVID-19 vaccine delivery in these same communities ensures that vaccination initiatives reach communities equitably with a flexible model that considers each school district's specific needs. Examples of specific needs included offering verbal or written consent without the requirement for in-person guardian for student vaccination, opportunity for school-based vaccine education, and open scheduling to account for same day appointments.

These lessons learned provide a blueprint for ongoing communication and preparation for any future pandemics or other disaster preparedness. Key strategies include maintaining strong school health partnerships in all area school districts with a focus on building trust and authentic engagement. This engagement will require clearly identified roles and communication systems in case of an emergency in which a quick mobilization is paramount. This communication is inclusive of not only district and school staff, but also communication channels best utilized by the school district to reach students and their families for information dissemination. The cultivation of trusted school personnel and communication channels are integral to the partnerships that necessarily sit at the center of effective pandemic or other health emergency responses.

## Conclusion

Vaccinating children is a critical piece in the larger pandemic puzzle, and a community-based population health approach can be an effective method for accomplishing this goal. Schools are ideal community partners for hosting vaccination clinics for the school-age population. Using their facilities and transportation systems can simplify otherwise complicated access to care logistics. Additionally, leveraging schools' communication systems to advertise clinics, provide education, and combat vaccine misinformation proved invaluable.

As with all partnerships, success requires trust and tolerance of uncertainty; especially when working with an adolescent population. The importance of clear and consistent communication cannot be emphasized enough when transforming pre-existing partnerships to achieve common goals in a rapidly changing environment. The COVID-19 pandemic made clear that planning and relationship building between schools and healthcare institutions must take place well before the arrival of public health emergencies to effectively leverage resources and combat crises.

## Data availability statement

The original contributions presented in the study are included in the article/supplementary material, further inquiries can be directed to the corresponding author.

## Author contributions

SB and MI drafted and revised the initial and final manuscripts. DS reviewed and revised the original manuscript, edited it, and prepared the final manuscript for submission. HP created and revised the timeline and the original manuscript. SB, HP, and DS addressed first-round reviewer comments and made required revisions. SB addressed second-round reviewer comments and made required revisions. All authors approved the final manuscript as submitted and agreed to be accountable for all aspects of the work.
